# The correlations between C-reactive protein and MRI-detected inflammation in patients with axial spondyloarthritis: a systematic review and meta-analysis

**DOI:** 10.1007/s10067-023-06658-w

**Published:** 2023-06-19

**Authors:** Haoran Tian, Ting Li, Yuanqiong Wang, Hongjuan Lu, Li Lin, Xin Wu, Huji Xu

**Affiliations:** 1 Department of Rheumatology and Immunology, Shanghai Changzheng Hospital, Naval Medical University, Shanghai, 200003 China; 2 Peking-Tsinghua Center for Life Sciences, Tsinghua University, Beijing, 100084 China; 3 School of Clinical Medicine, Tsinghua University, Beijing, 100084 China

**Keywords:** Axial spondyloarthritis, C-reactive protein, Disease activity, Magnetic resonance imaging

## Abstract

**Background:**

C-reactive protein (CRP) and magnetic resonance imaging (MRI) are widely used to monitor inflammation in patients with axial spondyloarthritis (axSpA), but the relationship between CRP and MRI-detected inflammation is incompletely understood. The present study was undertaken to assess correlations between CRP and MRI-detected inflammation in axSpA.

**Materials and methods:**

A systematic literature search was performed (Medline, Embase, and Cochrane Library) to identify relevant studies concerning CRP and MRI-detected inflammation in axSpA patients. The MRI-detected inflammation was evaluated by MRI-based disease activity score (DAS). The correlation between CRP and MRI-based DAS was integrated by random-effect models.

**Results:**

Eighteen studies reported a total of 1392 axSpA patients which were included in this meta-analysis. CRP was significantly associated with spinal MR DAS (*r*=0.226, 95%CI [0.149, 0.291], *p*<0.001, *I*^2^=23%). We also found a moderate correlation between CRP change and spinal MR DAS change (*r*[ASspiMRI-a]=0.354, 95%CI [0.282, 0.422], *p*<0.001, *I*^2^=48%; *r*[SPARCC]=0.544, 95%CI [0.345, 0.701], *p*<0.001, *I*^2^=19%). CRP at baseline was negatively associated with improvement in spinal MR DAS (*r*= − 0.327, 95%CI [−0.397, −0.264], *p*<0.001, *I*^2^=0%). However, no significant association was found between CRP and sacroiliac joint (SIJ) MR DAS.

**Conclusions:**

In axSpA patients, CRP is associated with MRI-detected inflammation in the spine but not in SIJ. We speculate that CRP could be a reasonable index to reflect spinal inflammation. Therefore, we suggest it is not essential to repeat spinal MRI in a short term, while SIJ MRI may be necessary to provide additional information on inflammation.

**Table:** 

**Key Points** *• CRP is associated with MRI-detected inflammation in the spine but not in sacroiliac joints*. *• CRP at baseline was negatively associated with improvement in spinal MR DAS*. *• It was not essential to repeat spinal MRI frequently, while SIJ MRI may be necessary to provide additional information on inflammation*.

**Supplementary Information:**

The online version contains supplementary material available at 10.1007/s10067-023-06658-w.

## Introduction

AxSpA is an inflammatory rheumatic disease of unknown etiology characterized by damages primarily in the axial skeleton, mainly in the SIJ and spreading to the whole spine. In previous studies, the prevalence of axSpA in different populations ranged from 0.32 to 1.4% [1]. The most typical manifestations of patients are chronic low back pain, morning stiffness, and fatigue. Pain, reduced mobility, and potential spinal deformity are caused by inflammation and structural damage.

Inflammation is a critical early step in osteoproliferation and structural remodeling [1]. The ultimate goals of axSpA treatment are to control inflammation, reduce disease activity, prevent radiographic progression, and maintain physical function [2]. So how to evaluate inflammation is of critical importance. However, to date, a broadly accepted tool to detect inflammation in axSpA is lacking. The basic so-called objective signs of inflammation, which have generally been recommended by various guidelines, included CRP and MRI. CRP is an acute-phase reactant and plays a prominent role in monitoring patients with axSpA [3]. Owing to its simplicity, repeatability, and reliability, CRP fulfills the “OMERACT filter” as a relevant outcome measurement in axSpA [3], whereas there are still some debates as to whether CRP is a valid indicator of inflammation [1, 4]. Some studies reported that CRP might not be elevated in active axSpA [5, 6]. In the past decade, the use of MRI has brought our vision into a new phase [7, 8]. MRI studies have contributed to detecting spinal and SIJ inflammation, even minor fluid collections such as bone marrow edema (BME) [9]. MR DAS provided a semi-quantitative measure to evaluate the spinal/SIJ inflammation in axSpA, including the Spondyloarthritis Research Consortium of Canada (SPARCC) [10, 11], the Ankylosing Spondylitis spine Magnetic Resonance Imaging-activity (ASspiMRI-a) [12], and the Berlin method [13]. Ample evidence suggests that MR DAS provides additional information on top of clinical and biochemical assessments [14]. Despite minor differences between these methods, all showed comparable discriminatory capacity and good sensitivity to change [2]. For the assessment of inflammation in SIJ, the most widely used scoring systems for quantification are the Berlin score and the SPARCC score [15]. As for the evaluation of spinal inflammation, all three scoring systems are commonly used. Although the contribution of MRI to our understanding of axSpA is indisputable [7, 8], MRI is time-consuming and expensive, which limits its clinical application. This has prompted extensive investigation of the correlation between CRP and MRI.

The relationship between MR DAS and CRP is incompletely understood. Some studies indicated weak or inconsistent correlations between CRP and MRI findings [16, 17]; BME could be detected by MRI in a sizable proportion (78.9%) of CRP-negative axSpA patients [18]. Other studies reported that CRP correlated with MR DAS, and a decrease in CRP was related to the improvement in MR DAS [19]. Taken together, the relationship between CRP and MRI-detected inflammation in patients with axSpA remains nebulous.

Considering the conflicting study results, we conducted a systematic review and meta-analysis to determine the correlation between CRP and MRI findings in patients with axSpA. To the best of our knowledge, this is the first meta-analysis to analyze the correlation between CRP and MRI, which may improve clinicians’ understanding of inflammation monitoring in axSpA patients.

## Methods

### Search strategy and study selection

This meta-analysis was conducted according to the guidelines of the Preferred Reporting Items for Systematic Reviews and Meta-Analysis (PRISMA) statement [20] (shown in Supplementary Table S1). PubMed, Cochrane, and Embase were searched for studies assessing CRP and MRI in axSpA patients from inception to 17 December 2020. Medical Subject Headings (MeSH) terms “Spondylitis, Ankylosing,” “C-Reactive Protein,” “Magnetic Resonance Imaging,” and related free text terms were used for the search. Besides, the reference lists of the obtained articles were scanned manually to identify additional relevant articles. The detailed search strategy is shown in Supplementary Data S1. After removing duplicate references, two reviewers (HRT and TL) screened titles and abstracts independently. Disagreements between reviewers were resolved by a discussion with a third reviewer (YQW) about eligibility. We registered the study protocol in the International Prospective Register of Systematic Reviews (PROSPERO: CRD42021251256) database.

**Fig. 1:**
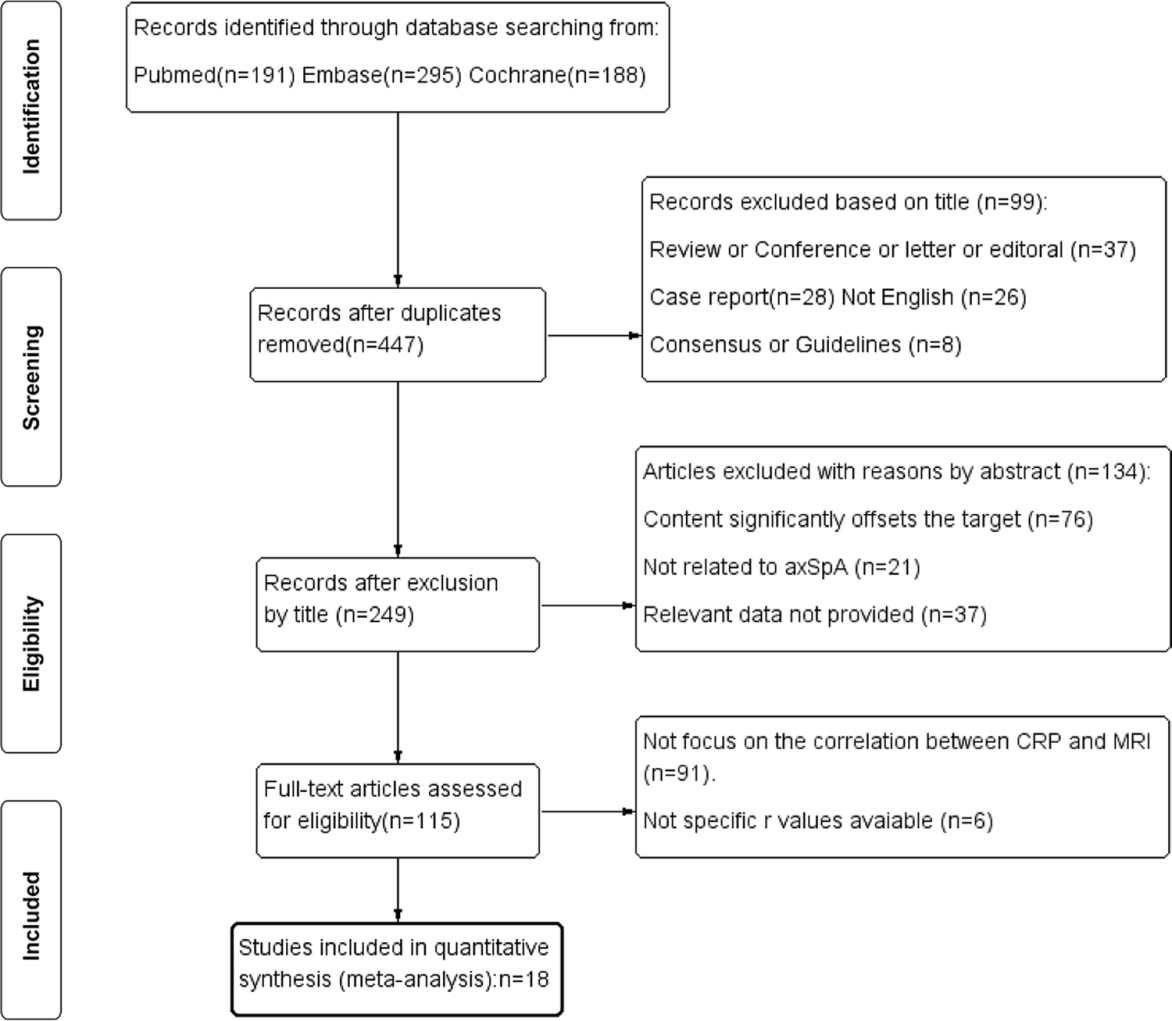
Flow chart describing the systematic search and study selection

The included studies were subjected to the following inclusion criteria: (1) all participants were adult patients (not less than 18 years old) with axSpA who met either the Modified New York criteria [21] or the Assessment of SpondyloArthritis international Society (ASAS) criteria [22]; (2) the results of correlation analysis between MR DAS and CRP levels were performed. The excluded criteria were manuscripts not (yet) published as original studies; opinion or discussion papers; not English; and no subject-related data could be extracted. Other exclusion criteria and paper screening processes are shown in Fig. 1.

### Risk of bias assessment and data extraction

Two authors (HRT and TL) independently assessed the risk of bias in this study. The QUADAS-2 tool for the Quality Assessment of Diagnostic Accuracy Studies includes four sections: patient selection, index test, reference standard, flow and timing [23]. Differences in assessment can be discussed. If consensus cannot be reached, a third reviewer (YQW) will rule. The risk of bias evaluation of this study is detailed in Supplementary Figure S1.

The results of data extraction by two reviewers (HRT and TL) from the first ten studies were identical, so the remaining fifteen articles were finished by one of the reviewers (HRT), and the other one was responsible for proofreading (TL). The contents of the data extraction include study identification (first author, journal, year of publication), number of patients, assessed joints (SIJ or spine), MRI semi-quantitative scoring method, therapy, MRI scanning intervals, correlation coefficient, and *p*-value of the correlation between MR DAS and clinical features. If there was no specific correlation coefficient (*r*-value) but only a *p*-value, we would send an email to ask the author for data.

### Statistical analysis

Heterogeneity between studies was assessed using *I*^2^ statistics (*I*^2^ <30% = low heterogeneity; 30–60% = moderate heterogeneity; >60% = high heterogeneity) [24]. Whenever heterogeneity was high (*I*^2^ >50%), random-effect models were used [25]. Subgroup analyses were performed according to different sites of MRI (SIJ or spine) and different scoring methods (SPARCC, Berlin, ASspiMRI). The correlation coefficient (*r*-value) extracted from each study was converted using Fisher’s *Z* transformation, and the conversion formulas were shown in Formulas 1, 2, and 3.

1 $${\mathrm{Fisher}}^{\hbox{'}}\mathrm{s}\ Z=0.5\times \ln \sqrt{\frac{1+r}{1-r}}$$

2 $${V}_z=\frac{1}{n-3}$$

3 $$\mathrm{SE}=\sqrt{V_z}$$

4 $${r}_{\mathrm{summary}}=\frac{e^{2Z\mathrm{summary}\ \mathrm{Fisher}\hbox{'}\mathrm{s}\ Z}-1}{e^{2Z\mathrm{summary}\ \mathrm{Fisher}\prime \mathrm{s}\ Z}+1}$$

The converted Fisher’s *Z* value and SE (standard error) value were entered into the ReVman software (version ReVman 5.4); the inverted variance method was used to obtain the summary Fisher’s *Z* value (including 95% confidence interval). *p* < 0.05 was considered statistically significant, and then the summary *r* value was calculated according to Formula 4.

## Results

### Study characteristics

Through the screening of 447 studies, there were 24 studies concerning the association between CRP and MR DAS. Six studies [26–31] were excluded from the meta-analysis due to the absence of a specific *r*-value between CRP change and MR DAS change. Eighteen studies were included in this meta-analysis. There were 11 studies [16, 17, 19, 32–39] involving the correlation between clinical features of CRP and MR DAS, 3 studies [19, 38, 40] analyzing the predictive effects of baseline CRP on MR DAS change, and 10 studies [16, 19, 38, 40–46] focusing on the relationship between CRP change and MR DAS change. We included 6 cross-sectional studies [32–35, 37, 39], 2 clinical trials [30, 43], 3 cohort studies [16, 28, 31], and 12 randomized controlled trials (RCTs) [17, 19, 26, 27, 29, 36, 38, 40, 41, 44–46]. Maksymowych’s research [42] included a cross-sectional study and a cohort study. Most of the studies judged by two reviewers were low-risk, except for 2 cross-sectional studies [33, 37] and 1 cohort study [35] (shown in Supplementary Figure S1).

### Meta-analysis

#### Correlation between CRP and MR DAS

**Fig. 2:**
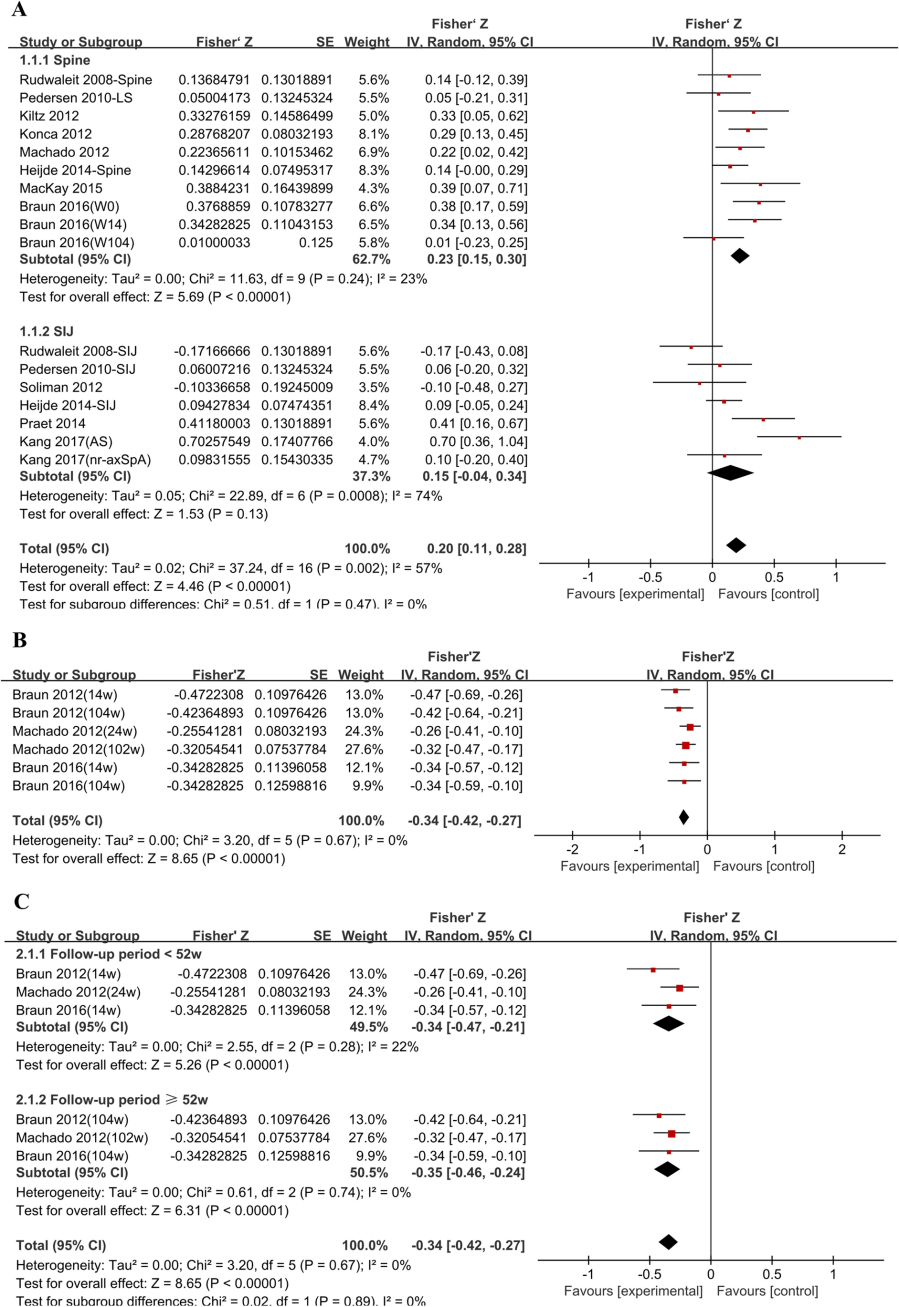
**A** Correlation between CRP and MR DAS. **B** Correlation between baseline CRP and MR DAS change. **C** Subgroup correlation between baseline CRP and MR DAS change

A total of 1325 patients were included in the meta-analysis of CRP/MR DAS correlation. Subgroup analysis was conducted based on different MRI sites (842 patients in the spine subgroup, 483 patients in the SIJ subgroup). The correlation coefficient in the spine subgroup was calculated based on the data extracted from 8 studies [16, 17, 19, 32, 33, 36–38] (shown in Table 1). There was a modest correlation between CRP and spinal MR DAS (*r*=0.226, 95%CI [0.149, 0.291], *p* <0.001, *I*^2^=23%). In the SIJ subgroup, the pooled *r* of 6 studies [16, 17, 34–36, 39] indicated no statistically significant (*r*=0.149, 95%CI [−0.040, 0.327], *p*=0.130, *I*^2^=74%) (shown in Fig. 2A).

**Table 1:** Correlation between CRP and MR DAS

Study	Scoring method	Location	Number	CRP	ESR	BASDAI	ASDAS	BASMI	BASFI
Rudwaleit 2008 (17)	Berlin	Spine	62	0.136 (NS)	0.195 (NS)	−0.033 (NS)	–	0.235 (NS)	−0.163 (NS)
		SIJ	62	−0.170 (NS)	−0.070 (NS)	0.001 (NS)	–	−0.499 (0.001)	−0.162 (NS)
Pedersen 2010 (16)	Berlin	SIJ	56–60	0.060 (NS)	–	−0.230 (NS)	−0.140 (NS)	–	–
		LS	56–60	0.050 (NS)	–	−0.410 (*p*<0.01)	−0.300 (<0.05)	–	–
Konca 2012 (33)	ASspiMRI-a	Spine	50	0.321 (0.023)	0.244 (0.088)	−0.020 (0.915)	–	0.396 (0.004)	0.222 (0.122)
Machado 2012 (19)	M-ASspiMRI-a	spine	158	0.280 (<0.001)	–	−0.090 (0.174)	0.160 (0.016)	–	–
Kiltz 2012 (32)	Berlin	Spine	100	0.220 (0.030)	–	NS	NS	–	–
Soliman 2012 (34)	BME score	SIJ	30	−0.103 (0.589)	0.256 (0.290)	0.119 (0.537)	–	−0.513 (0.004)	−0.267 (0.161)
Heijde 2014 (36)	SPARCC	SIJ	182	0.094 (NS)	–	−0.187 (0.010)	0.022 (NS)	–	−0.105 (NS)
		Spine	181	0.142 (NS)	–	−0.030 (NS)	0.123 (NS)	–	0.043 (NS)
Praet 2014 (35)	SPARCC	SIJ	62	0.390 (0.002)	–	0.100 (0.440)	0.350 (0.007)	–	–
MacKay 2015 (37)	SPARCC	SIJ	40	NS	NS	0.120 (0.470)	0.120 (0.460)	–	–
		Spine	40	0.370 (0.020)	0.380 (0.020)	0.160 (0.330)	0.280 (0.080)	–	–
Braun2016 (38)	ASspiMRI-a	Spine	89	w0:0.360 (0.009)	–	–	–	–	–
		Spine	85	w14:0.330 (0.036)	–	–	–	–	–
		Spine	67	w104:0.010 (1.000)	–	–	–	–	–
Kang 2017 (39)	SPARCC	SIJ	36 (nr-axSpA)	0.606 (<0.001)	0.576 (0.001)	0.001 (0.995)	0.453 (0.006)	–	–
		SIJ	45 (AS)	0.098 (0.523)	0.066 (0.668)	0.059 (0.698)	0.163 (0.285)	–	–

The Spearman test for rank correlation is used for test of correlation; values are correlation coefficients (rho), if not otherwise indicated. *p*-values indicate the level of statistical significance. *AS*, ankylosing spondylitis; *LS*, lumbar spine; *ASDAS*, Ankylosing Spondylitis Disease Activity Score; *ASspiMRI-a*, ankylosing spondylitis spine MRI score for activity; *M-ASspiMRI-a*, modified ASspiMRI-a; *BASDAI*, Bath Ankylosing Spondylitis Disease Activity Score; *CRP*, C-reactive protein; *ESR*, erythrocyte sedimentation rate; *MRI*, magnetic resonance imaging; *NS*, not statistically significant; *SIJ*, sacroiliac joints; *SPARCC*, Spondyloarthritis Research Consortium of Canada Scoring System; –, not done

#### Correlation between baseline CRP and MR DAS change

There were 3 RCTs on the relationship between baseline CRP and spinal MR DAS change [19, 38, 40]. Data on the correlation between baseline CRP and SIJ MR DAS was not available. A total of 655 patients were included in the data synthesis (shown in Table 2). The result of the summary correlation showed that baseline CRP was negatively associated with spinal MR DAS change (*r* = −0.327, 95%CI [−0.397, −0.264], *p* <0.001, *I*^2^=0%) (shown in Fig. 2B). Subgroup analysis was conducted based on the follow-up period (<52 weeks or ≥52 weeks). A significant association was found in both short period subgroup (*r* = −0.319, 95%CI [−0.414, −0.217], *p*<0.001, *I*^2^=22%) and long period subgroup (*r* = −0.336, 95%CI [−0.430, −0.235], *p*<0.001, *I*^2^=0%) (shown in Fig. 2C).

**Table 2:** Correlation between baseline CRP and MR DAS change

Study	Scoring method	Location	Number	Therapy	Scan interval	CRP	BASDAI	ASDAS	BASFI
Braun 2012 (40)	ASspiMRI-a	Spine	86	GOL	14w	−0.440 (0.001)	−0.060 (NS)	−0.300 (0.015)	0.010 (NS)
		Spine	86	GOL	104w	−0.400 (<0.001)	−0.160 (NS)	−0.330 (0.010)	−0.210 (NS)
Machado 2012 (19)	M-ASspiMRI-a	Spine	158	IFX/PBO	24w	−0.250 (0.002)	0.120 (0.132)	−0.140 (0.076)	–
		Spine	179	IFX/PBO	102w	−0.310 (0.001)	0.140 (0.063)	−0.150 (0.044)	–
Braun 2016 (38)	ASspiMRI-a	Spine	80	GOL	14w	−0.330 (0.046)	–	–	–
		Spine	66	GOL	104w	−0.330 (0.018)	–	–	–

The Spearman test for rank correlation is used for test of correlation; values are correlation coefficients (rho), if not otherwise indicated. *p*-values indicate the level of statistical significance. *AS*, ankylosing spondylitis; *ASDAS*, Ankylosing Spondylitis Disease Activity Score; *ASspiMRI-a*, ankylosing spondylitis spine MRI score for activity; *M-ASspiMRI-a*, modified ASspiMRI-a; *BASDAI*, Bath Ankylosing Spondylitis Disease Activity Score; *CRP*, C-reactive protein; *ESR*, erythrocyte sedimentation rate; *MRI*, magnetic resonance imaging; *NS*, not statistically significant; *SPARCC*, Spondyloarthritis Research Consortium of Canada Scoring System; *GOL*, golimumab; *IFX*, infliximab; *PBO*, placebo; –, not done

#### Correlation between CRP change and MR DAS change

**Fig. 3:**
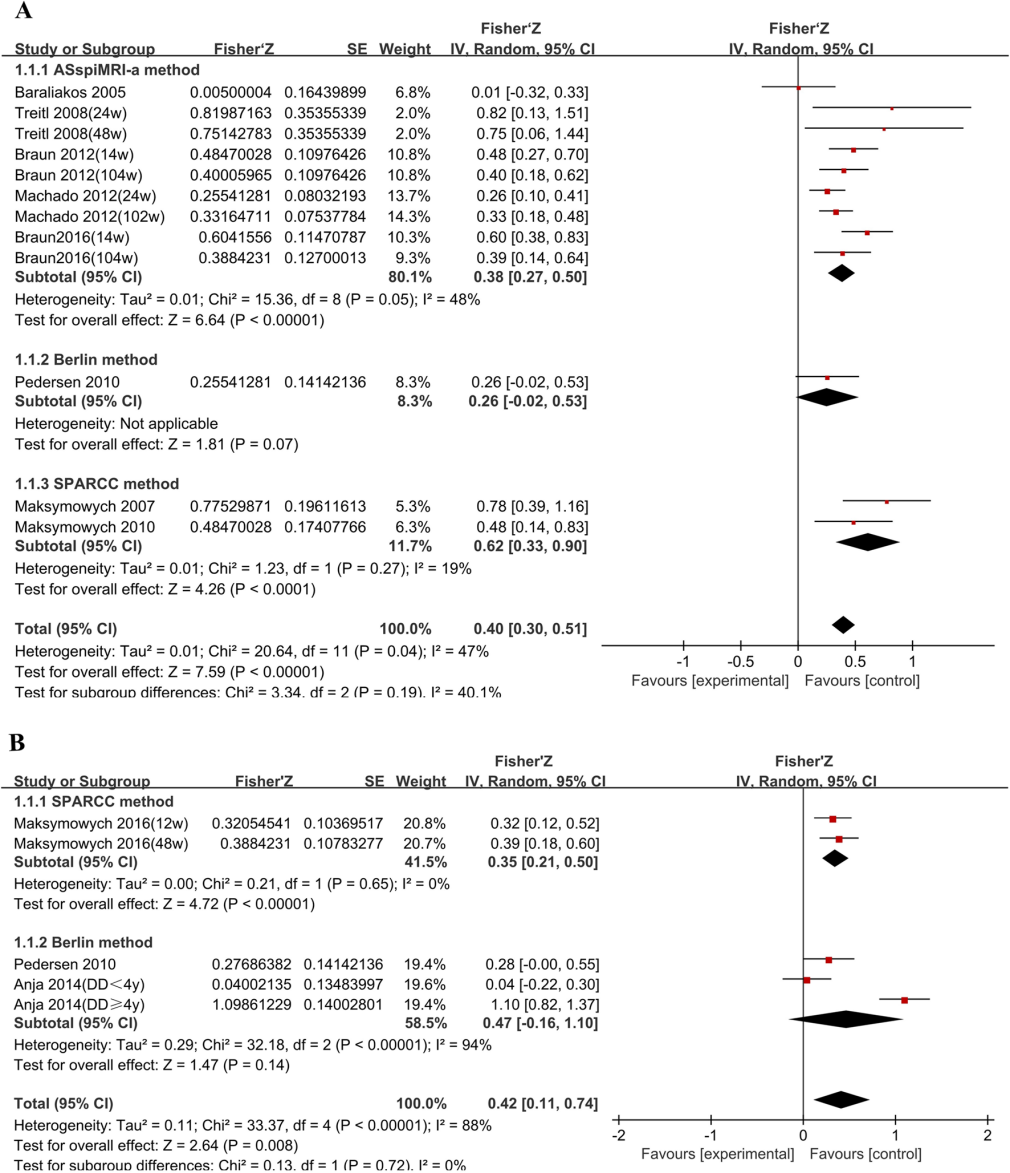
**A** Correlation between CRP change and MR DAS change (spine). **B** Correlation between CRP change and MR DAS change (SIJ)

As for the relationship between CRP change and spinal MR DAS change, 8 studies [16, 19, 38, 40–44] and 833 patients were included (shown in Table 3). CRP change was significantly associated with spinal MR DAS change (*r*=0.380, 95%CI [0.310, 0.450], *p*<0.001, *I*^2^=50.6%). Subgroup analysis was conducted based on different scoring methods (SPARCC, ASspiMRI-a, Berlin). We found a modest correlation in the ASspiMRI-a subgroup (*r*=0.354, 95%CI [0.282, 0.422], *p*<0.001, *I*^2^=48%) and moderate association in the SPARCC subgroup (*r*=0.544, 95%CI [0.345, 0.701], *p*<0.001, *I*^2^=19%) (shown in Fig. 3A).

**Table 3:** Correlation between CRP change and MR DAS change

Study	Scoring method	Location	*N*	Therapy	Scan interval	CRP	ESR	BASDAI	ASDAS	BASMI	BASFI
Baraliakos 2005 (41)	ASspiMRI-a	Spine	40	ETN/PBO	48w	0.005 (NS)	0.016 (NS)	0.110 (NS)	–	–	–
Lambert 2007 (26) #	SPARCC	Spine	38	ADA	12w	*p*=0.018	–	NS	–	NS	–
		SIJ	38	ADA	12w	*p*=0.590	–	NS	–	NS	–
Maksymowych 2007 (42)	SPARCC	Spine	29	IFX/PBO	12/24w	0.650 (<0.001)	–	0.340 (NS)	–	–	–
Visvanathan 2008 (27) #	ASspiMRI-a	Spine	279	IFX/PBO	24w	*p*<0.001	–	—	–	–	–
Treitl 2008 (43)	ASspiMRI-a	Spine	11	IFX	24w	0.675 (<0.023)	–	0.831 (<0.001)	–	–	–
		Spine	11	IFX	48w	0.636 (<0.036)	–	0.369 (<0.001)	–	–	–
Marzo-Ortega 2009 (28) #	Leeds	Spine	76	NSAIDs/SSZ	12m	NS	–	NS	–	–	NS
Pedersen 2010 (16)	Berlin	SIJ	47–53	TNFa	22w	0.270 (NS)	–	0.310 (NS)	0.460 (<0.010)	–	–
		LS	47–53	TNFa	22w	0.250 (NS)	–	−0.050 (NS)	0.220 (NS)	–	–
Maksymowych 2010 (44)	SPARCC	Spine	36	IFX or PBO	12w	0.450 (0.012)	0.570 (0.001)	0.250 (NS)	–	–	0.160 (NS)
Song 2011 (29) #	Modified method	SIJ	76	ETN/SSZ	48w	NS	–	*p*=0.040	–	–	*p*=0.007
		Spine	76	ETN/SSZ	48w	NS	–	NS	–	–	NS
Braun 2012 (40)	ASspiMRI-a	Spine	86	GOL	14w	0.450 (<0.001)	–	0.260 (<0.050)	0.350 (0.004)	–	0.190 (NS)
	ASspiMRI-a	Spine	86	GOL	104w	0.380 (<0.010)	–	0.110 (<NS)	0.220 (NS)	–	0.050 (NS)
Machado 2012 (19)	Modified ASspiMRI-a	Spine	158	IFX/PBO	24w	0.250 (0.002)	–	0.140 (0.090)	0.220 (0.006)	–	–
		Spine	179	IFX/PBO	102w	0.320 (<0.001)	–	0.140 (0.057)	0.230 (0.002)	–	–
Karpitschka 2013 (30) #	Lesions count	SIJ	10	ETN	52w	NS	–	0.009	–	–	NS
		Spine	10	ETN	52w	NS	–	0.001	–	–	0.003
		Enthesitis	10	ETN	52w	NS	–	NS	–	–	NS
Anja 2014 (45)	Berlin	SIJ (DD <4)	58	ETN/ADA	48w	0.040 (0.900)	–	0.370 (0.010)	–	–	0.400 (0.010)
		SIJ (DD ≥4)	54	ETN/ADA	48w	0.800 (0.010)	–	0.120 (0.500)	–	–	0.100 (0.700)
Maksymowych 2016 (46)	SPARCC	SIJ	94–97	ETN	12w	0.310 (<0.010)	–	0.270 (<0.010)	0.350 (<0.001)	0.070 (NS)	0.170 (NS)
		SIJ	88–90	ETN	48w	0.370 (<0.001)	–	0.420 (<0.001)	0.580 (<0.001)	0.140 (NS)	0.350 (<0.001)
Braun2016 (38)	ASspiMRI-a	Spine	79	GOL	14w	0.540 (<0.001)	–	–	–	–	–
		Spine	65	GOL	104w	0.370 (0.045)	–	–	–	–	–
Tang 2018 (31) #	SPARCC	SIJ	33	NSAIDs	24/48w	NS	NS	–	NS	–	–

The Spearman test for rank correlation is used for test of correlation; values are correlation coefficients (rho), if not otherwise indicated. *p*-values indicate the level of statistical significance. *ASDAS*, Ankylosing Spondylitis Disease Activity Score; *ASspiMRI-a*, ankylosing spondylitis spine MRI score for activity; *BASDAI*, Bath Ankylosing Spondylitis Disease Activity Score; *NS*, not statistically significant; *SPARCC*, Spondyloarthritis Research Consortium of Canada Scoring System; *LS*, lumbar spine; *N*, number; *DD*, disease duration; *IFX*, infliximab; *SSZ*, sulfasalazine; *ADA*, adalimumab, *ETN*, etanercept; *GOL*, golimumab; *IFN*, infliximab; *PBO*, placebo; *NSAIDs*, non-steroidal anti-inflammatory drugs; *y*, year; *m*, month; *w*, week; –, not done

#specific *r* values were not available

As for the relationship between CRP change and SIJ MR DAS change, 3 studies [16, 45, 46] and 340 patients were included (shown in Table 3). Subgroup analysis was conducted based on different scoring methods (SPARCC, Berlin). We found no association in the Berlin subgroup (*p*=0.140) and modest correlation in the SPARCC subgroup (*r*=0.336, 95%CI [0.207, 0.462], *p*<0.001, *I*^2^=0%) (shown in Fig. 3B).

## Discussion

AxSpA is a chronic rheumatic disease that affects the function of axial and peripheral joints [47]. Inflammation is a critical early step in new syndesmophyte formation and structural remodeling in axSpA [48]. Sustained inflammation leads to irreversible skeleton damage and poor physical function and therefore should be monitored critically [49]. CRP and MRI are now widely used as objective tools to evaluate inflammation in axSpA. We conducted a systematic review and meta-analysis to analyze the correlation between CRP and MRI findings in patients with axSpA.

Our results illustrated that CRP correlated with spinal MR DAS. We found a modest association between CRP and spinal MR DAS (*r*=0.226, *I*^2^=23%), and a moderate correlation between CRP change and spinal MR DAS change (ASspiMRI, *r*=0.354, *I*^2^=48%; SPARCC, *r*=0.544, *I*^2^=19%). Although CRP is closely related to inflammation, some studies reported that CRP might not be elevated in active axSpA [6, 18]. MRI studies have contributed to detecting spinal inflammation, even minor fluid collections such as BME. However, it is not feasible in most settings and is too costly to repeat MRIs frequently [9]. Given the lack of evidence that obtaining an MRI in stable patients improves clinical outcomes, the American College of Rheumatology (ACR) and the Spondylitis Association of America (SAA) recommended against obtaining an MRI regularly in axSpA [50]. Our results confirmed the correlation between CRP and spinal MR DAS. We speculated that CRP was a valid index to evaluate spinal inflammation in axSpA patients. Considering the feasibility of daily clinical practice, CRP is a reliable indicator for evaluating spinal inflammation.

Although our results illustrated the relationship between CRP and spinal MR DAS, we did not find a statistical correlation between CRP and SIJ MR DAS (*r*=0.149, *I*^2^=74%). It was reported that BME could be detected by SIJ MRI in a sizable proportion of CRP-negative SpA patients [18]. According to our results, MRI may provide additional information on SIJ inflammation in axSpA. We recommend SIJ MRI follow-up, especially in patients with unrelieved clinical manifestations such as low back pain, stiffness, and fatigue. Considering the high heterogeneity of studies included in analyzing the correlation between CRP and SIJ MRI, we look forward to more studies with relatively low heterogeneity to be included in the future.

We also identified a negative correlation between baseline CRP and spinal MRI improvement (*r* = −0.327, *I*^2^=0%). Our results provided valuable information that CRP may predict disease progression in axSpA. We speculated that residual inflammation might exist in axSpA patients with elevated CRP at baseline. In line with our hypothesis, it was reported that CRP could predict subsequent structural remodeling [51–53]. Consequently, we suggested that patients with elevated CRP at baseline needed more robust anti-inflammatory treatment or early initiation of biologicals. Long-term administration of biologics might be necessary for patients with high CRP levels at baseline.

To our knowledge, this is the first systematic review with meta-analysis to investigate the correlation between CRP and MR DAS in axSpA patients. Most studies included in our meta-analysis showed low-to-moderate heterogeneity (shown in Figs. 2 and 3), and some studies (those analyzed for baseline CRP and spinal MR DAS change) had even no heterogeneity (shown in Fig. 2). However, a few studies (those analyzed for CRP and SIJ MR DAS, CRP change, and SIJ MR DAS change) showed high heterogeneity. This may be due to differences in scoring methods and disease duration of patients among the studies. We therefore used subgroup analysis (e.g., SPARCC method versus Berlin method) and random-effect models to reduce heterogeneity. Our study confirmed that CRP is not only a valid indicator for spinal inflammation, but also a predictive parameter for disease course. Our work shed new light on the added value of CRP in diagnosis and disease monitoring.

It should be noted that this meta-analysis also has several limitations. First, different scoring methods are widely used to quantify inflammation in axSpA, and the issue remains about which could be more related to pathological manifestation. It is disputable whether SIJ or spinal inflammation assessment requires all slices/disco-vertebral units (DVUs) or the most heavily involved slices/DVUs. Hence, any scoring method can only be used as a semi-quantitative tool rather than a gold standard. Second, there should be an extensive focus on the disease duration. Anja et al. [45] reported that MR DAS change in SIJ was associated with CRP change in patients with disease duration longer than 4 years. However, there are not enough studies to stratify patients and sufficient evidence may be needed to validate it. Finally, we did not add study types to the inclusion criteria due to the limited number of studies concerning CRP and MRI in axSpA, which led to high heterogeneity in the correlation analysis between CRP and SIJ MR DAS.

In summary, CRP could be a reasonable index to reflect spinal inflammation, while SIJ MRI may be necessary to repeat providing additional information in the short term.

## Conclusions

This systematic review and meta-analysis preliminarily explored the relationship between CRP and MR DAS. The available evidence is in favor of CRP as an indicator and predictive parameter for spinal inflammatory lesions in axSpA. Nevertheless, SIJ MRI seems to be indispensable in disease monitoring.

## Supplementary information

ESM 1

ESM 2

ESM 3

## Data Availability

Not applicable

## Abbreviations

CRP: C-reactive protein
axSpA: Axial spondyloarthritis
MRDAS: Magnetic resonance imaging-based disease activity score
MRI: Magnetic resonance imaging
SPARCC: Spondyloarthritis Research Consortium of Canada
ASspiMRI-a: Ankylosing Spondylitis spine Magnetic Resonance Imaging-activity
BME: Bone marrow edema
SIJ: Sacroiliac joints
