# Supplementary figures and images for: The correlations between C-reactive protein and MRI-detected inflammation in patients with axial spondyloarthritis: a systematic review and meta-analysis
Source: Clin Rheumatol. 2023 Jun 19;42(9):2397–407. doi: 10.1007/s10067-023-06658-w (PMC10412674; doi:10.1007/s10067-023-06658-w)

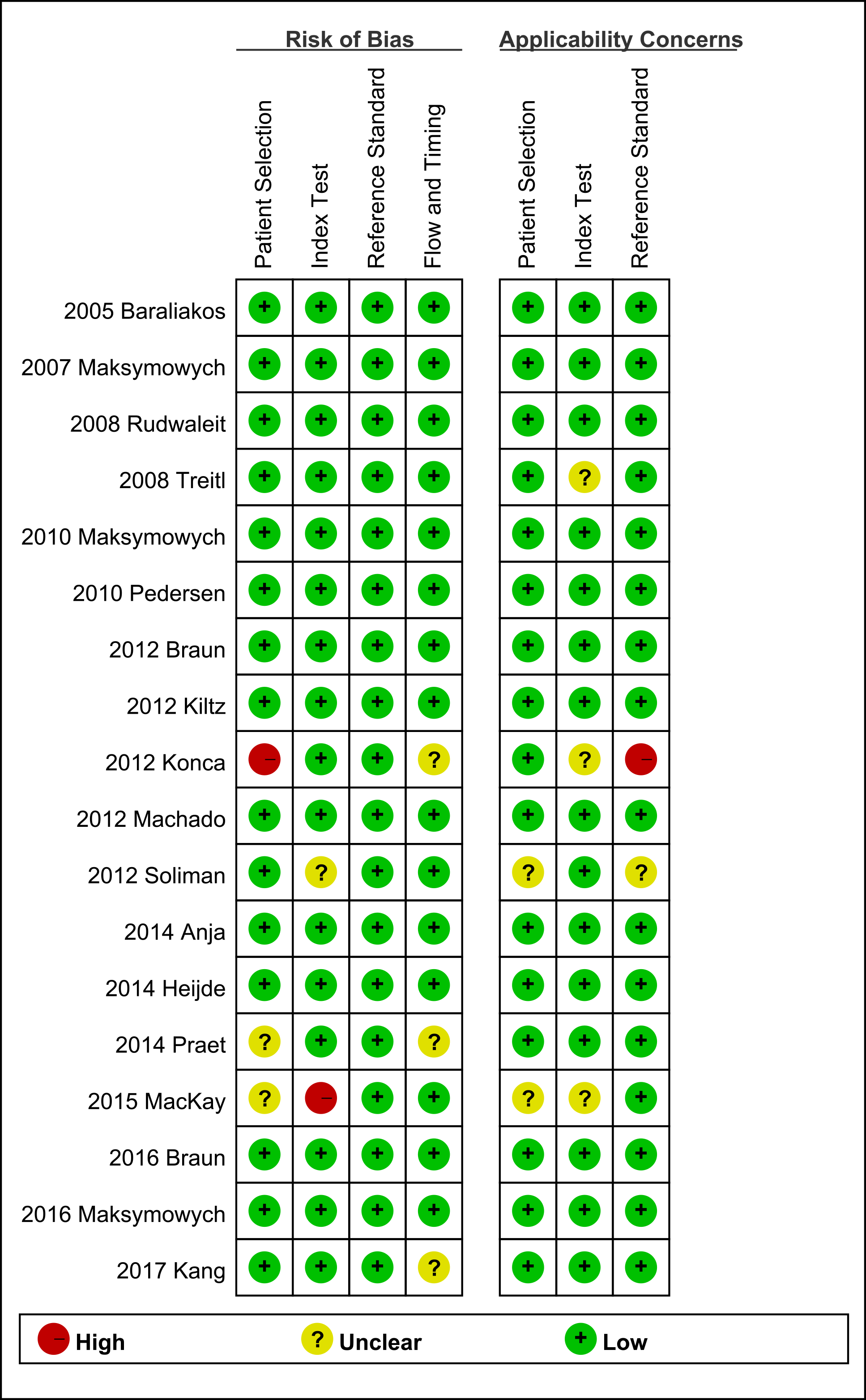

Supplement: Supplementary file 2 — ESM 2 [file 10067_2023_6658_MOESM2_ESM.tif]
