# Supplementary material for: The correlations between C-reactive protein and MRI-detected inflammation in patients with axial spondyloarthritis: a systematic review and meta-analysis
Source: Clin Rheumatol. 2023 Jun 19;42(9):2397–407. doi: 10.1007/s10067-023-06658-w (PMC10412674; doi:10.1007/s10067-023-06658-w)
Supplement: Supplementary file 3 — ESM 3 [file 10067_2023_6658_MOESM3_ESM.pdf]

Search Strategy：

PUBMED：

(1) MeSH：**Spondylitis, Ankylosing**

Entry Terms:

- Spondyloarthritis Ankylopoietica
- Ankylosing Spondylarthritis
- Ankylosing Spondylarthritides
- Spondylarthritides, Ankylosing
- Spondylarthritis, Ankylosing
- Ankylosing Spondylitis
- Spondylarthritis Ankylopoietica
- Bechterew Disease
- Bechterew's Disease
- Bechterews Disease
- Marie-Struempell Disease
- Marie Struempell Disease
- Rheumatoid Spondylitis
- Spondylitis, Rheumatoid
- Spondylitis Ankylopoietica
- Ankylosing Spondyloarthritis
- Ankylosing Spondyloarthritides
- Spondyloarthritides, Ankylosing
- Spondyloarthritis, Ankylosing

(2) MeSH：**C-Reactive Protein:**

Entry Terms:

- C Reactive Protein
- hsCRP
- High Sensitivity C-Reactive Protein
- High Sensitivity C Reactive Protein
- hs-CRP

(3) MeSH：**Magnetic Resonance Imaging**

Entry Terms:

- Imaging, Magnetic Resonance
- NMR Imaging
- Imaging, NMR
- Tomography, NMR
- Tomography, MR
- MR Tomography
- NMR Tomography
- Steady-State Free Precession MRI
- Steady State Free Precession MRI
- Zeugmatography
- Imaging, Chemical Shift
- Chemical Shift Imagings
- Imagings, Chemical Shift
- Shift Imaging, Chemical
- Shift Imagings, Chemical
- Chemical Shift Imaging
- Magnetic Resonance Image
- Image, Magnetic Resonance
- Magnetic Resonance Images

- Resonance Image, Magnetic
- Magnetization Transfer Contrast Imaging
- MRI Scans
- MRI Scan
- Scan, MRI
- Scans, MRI
- Tomography, Proton Spin
- Proton Spin Tomography
- fMRI
- MRI, Functional
- Functional MRI
- Functional MRIs
- MRIs, Functional
- Functional Magnetic Resonance Imaging
- Magnetic Resonance Imaging, Functional
- Spin Echo Imaging
- Echo Imaging, Spin
- Echo Imagings, Spin
- Imaging, Spin Echo
- Imagings, Spin Echo
- Spin Echo Imagings

(Magnetic Resonance Imaging) OR (Imaging, Magnetic Resonance) OR (NMR Imaging) OR (Imaging, NMR) OR (Tomography, NMR) OR (Tomography, MR) OR (MR Tomography) OR (NMR Tomography) OR (Steady-State Free Precession MRI) OR (Steady State Free Precession MRI) OR (Zeugmatography) OR (Imaging, Chemical Shift) OR (Chemical Shift Imagings) OR (Imagings,

Chemical Shift) OR (Shift Imaging, Chemical) OR (Shift Imagings, Chemical) OR (Chemical Shift Imaging) OR (Magnetic Resonance Image) OR (Image, Magnetic Resonance) OR (Magnetic Resonance Images) OR (Resonance Image, Magnetic) OR (Magnetization Transfer Contrast Imaging) OR (MRI Scans) OR (MRI Scan) OR (Scan, MRI) OR (Scans, MRI) OR (Tomography, Proton Spin) OR (Proton Spin Tomography) OR (fMRI) OR (MRI, Functional) OR (Functional MRI) OR (Functional MRIs) OR (MRIs, Functional) OR (Functional Magnetic Resonance Imaging) OR (Magnetic Resonance Imaging, Functional) OR (Spin Echo Imaging) OR (Echo Imaging, Spin) OR (Echo Imagings, Spin) OR (Imaging, Spin Echo) OR (Imagings, Spin Echo) OR (Spin Echo Imagings)

((1) AND (2)) AND (3)：

(((Spondylitis, Ankylosing) OR (Spondyloarthritis Ankylopoietica) OR (Ankylosing Spondylarthritis) OR (Ankylosing Spondylarthritides) OR (Spondylarthritides, Ankylosing) OR (Ankylosing Spondylitis) OR (Spondylarthritis Ankylopoietica) OR (Bechterew Disease) OR (Bechterew's Disease) OR (Bechterews Disease) OR (Marie-Struempell Disease) OR (Marie Struempell Disease) OR (Rheumatoid Spondylitis) OR (Spondylitis, Rheumatoid) OR (Spondylitis Ankylopoietica) OR (Ankylosing Spondyloarthritis) OR (Ankylosing Spondyloarthritides) OR (Spondyloarthritides, Ankylosing) OR (Spondyloarthritis, Ankylosing) OR (Spondyloarthritis) OR (axial SpA) OR (axial Spondyloarthritis) OR (axSpA) OR (Ankylosing Spondylitis)[Title/Abstract]) AND ((C-Reactive Protein) OR (C Reactive Protein) OR (hsCRP) OR (High Sensitivity C-Reactive Protein) OR (High Sensitivity C Reactive Protein)

OR (hs-CRP) OR (CRP))) AND ((Magnetic Resonance Imaging) OR (Imaging, Magnetic Resonance) OR (NMR Imaging) OR (Imaging, NMR) OR (Tomography, NMR) OR (Tomography, MR) OR (MR Tomography) OR (NMR Tomography) OR (Steady-State Free Precession MRI) OR (Steady State Free Precession MRI) OR (Zeugmatography) OR (Imaging, Chemical Shift) OR (Chemical Shift Imagings) OR (Imagings, Chemical Shift) OR (Shift Imaging, Chemical) OR (Shift Imagings, Chemical) OR (Chemical Shift Imaging) OR (Magnetic Resonance Image) OR (Image, Magnetic Resonance) OR (Magnetic Resonance Images) OR (Resonance Image, Magnetic) OR (Magnetization Transfer Contrast Imaging) OR (MRI Scans) OR (MRI Scan) OR (Scan, MRI) OR (Scans, MRI) OR (Tomography, Proton Spin) OR (Proton Spin Tomography) OR (fMRI) OR (MRI, Functional) OR (Functional MRI) OR (Functional MRIs) OR (MRIs, Functional) OR (Functional Magnetic Resonance Imaging) OR (Magnetic Resonance Imaging, Functional) OR (Spin Echo Imaging) OR (Echo Imaging, Spin) OR (Echo Imagings, Spin) OR (Imaging, Spin Echo) OR (Imagings, Spin Echo) OR (Spin Echo Imagings) OR (MRI)) NOT (review) NOT(case report)

191results

Embase：

('spondylarthritis'/exp OR 'arthritis, spine' OR 'axial spondyloarthritis' OR 'spine arthritis' OR 'spondylarthritis' OR 'spondyloarthritis' OR 'vertebral arthritis' OR 'vertebral osteo-arthritis' OR 'vertebral osteoarthritis' OR 'ankylosing spondylitis'/exp OR 'bechterew disease' OR 'ankylating spondylitis' OR 'ankylopoietic spondylarthritis' OR 'ankylopoietic spondylitis' OR 'ankylosing spine' OR 'ankylosing spondilitis' OR

'ankylosing spondylarthritis' OR 'ankylosing spondylarthrosis' OR 'ankylosing spondylitis' OR 'ankylosis spondylitis' OR 'ankylotic spondylitis' OR 'bekhterev disease' OR 'morbus bechterew' OR 'spinal ankylosis' OR 'spine ankylosis' OR 'spondylarthritis ankylopoietica' OR 'spondylarthritis ankylosans' OR 'spondylarthrosis ankylopoietica' OR 'spondylitis ankylopoetica' OR 'spondylitis ankylopoietica' OR 'spondylitis, ankylosing' OR 'spondyloarthritis ankylopoietica' OR 'vertebral ankylosis') AND ('c reactive protein'/exp OR 'c reactive protein' OR 'c reaction protein' OR 'c-reactive protein' OR 'creactive protein' OR 'crp' OR 'protein, c reactive' OR 'serum c reactive protein') AND ('nuclear magnetic resonance imaging'/exp OR 'mri' OR 'nmr imaging' OR 'imaging, magnetization transfer' OR 'magnetic resonance imaging' OR 'magnetic resonance tomography' OR 'magnetization transfer imaging' OR 'mr imaging' OR 'nuclear magnetic resonance imaging')

Embase Session Results (16 Dec 2020)

**No.**	**Query**	**Results**
**#6**	#2 AND #3 AND #4 AND ([article]/lim OR [article in press]/lim)	**295**
**#5**	#2 AND #3 AND #4	**872**
**#4**	'nuclear magnetic resonance imaging'/exp OR 'mri' OR 'nmr imaging' OR 'imaging, magnetization transfer' OR 'magnetic resonance imaging' OR 'magnetic resonance tomography' OR 'magnetization transfer imaging' OR 'mr imaging' OR 'nuclear magnetic resonance imaging'	**1061401**
**#3**	'c reactive protein'/exp OR 'c reactive protein' OR 'c reaction protein' OR 'c-reactive protein' OR 'creactive protein' OR	**231592**

No.	Query	Results
	'crp' OR 'protein, c reactive' OR 'serum c reactive protein'	
#2	'ankylosing spondylitis'/exp/mj OR 'bechterew disease'/mj OR 'ankylating spondylitis'/mj OR 'ankylopoietic spondylarthritis'/mj OR 'ankylopoietic spondylitis'/mj OR 'ankylosing spine'/mj OR 'ankylosing spondilitis'/mj OR 'ankylosing spondylarthritis'/mj OR 'ankylosing spondylarthrosis'/mj OR 'ankylosing spondylitis'/mj OR 'ankylosis spondylitis'/mj OR 'ankylotic spondylitis'/mj OR 'bekhterev disease'/mj OR 'morbus bechterew'/mj OR 'spinal ankylosis'/mj OR 'spine ankylosis'/mj OR 'spondylarthritis ankylopoietica'/mj OR 'spondylarthritis ankylosans'/mj OR 'spondylarthrosis ankylopoietica'/mj OR 'spondylitis ankylopoetica'/mj OR 'spondylitis ankylopoietica'/mj OR 'spondylitis, ankylosing'/mj OR 'spondyloarthritis ankylopoietica'/mj OR 'vertebral ankylosis'/mj OR 'spondylarthritis'/exp/mj OR 'arthritis, spine'/mj OR 'axial spondyloarthritis'/mj OR 'spine arthritis'/mj OR 'spondylarthritis'/mj OR 'spondyloarthritis'/mj OR 'vertebral arthritis'/mj OR 'vertebral osteo-arthritis'/mj OR 'vertebral osteoarthritis'/mj	**22678**
#1	'ankylosing spondylitis'/exp OR 'bechterew disease' OR 'ankylating spondylitis' OR 'ankylopoietic spondylarthritis' OR 'ankylopoietic spondylitis' OR 'ankylosing spine' OR 'ankylosing spondilitis' OR 'ankylosing spondylarthritis' OR 'ankylosing spondylarthrosis' OR 'ankylosing spondylitis' OR 'ankylosis spondylitis' OR 'ankylotic spondylitis' OR	**42550**

No.	Query	Results
	'bekhterev disease' OR 'morbus bechterew' OR 'spinal ankylosis' OR 'spine ankylosis' OR 'spondylarthritis ankylopoietica' OR 'spondylarthritis ankylosans' OR 'spondylarthrosis ankylopoietica' OR 'spondylitis ankylopoetica' OR 'spondylitis ankylopoietica' OR 'spondylitis, ankylosing' OR 'spondyloarthritis ankylopoietica' OR 'vertebral ankylosis' OR 'spondylarthritis'/exp OR 'arthritis, spine' OR 'axial spondyloarthritis' OR 'spine arthritis' OR 'spondylarthritis' OR 'spondyloarthritis' OR 'vertebral arthritis' OR 'vertebral osteo-arthritis' OR 'vertebral osteoarthritis'	

Cochrane：

Search Name: cochrane

Last Saved: 17/12/2020 11:41:46

Comment:

ID Search

#1 MeSH descriptor: [Spondylitis, Ankylosing] this term only

#2 ((Spondylitis, Ankylosing) OR (Spondyloarthritis Ankylopoietica) OR (Ankylosing Spondylarthritis) OR (Ankylosing Spondylarthritides) OR (Spondylarthritides, Ankylosing) OR (Ankylosing Spondylitis) OR (Spondylarthritis Ankylopoietica) OR (Bechterew Disease) OR

(Bechterew's Disease) OR (Bechterews Disease) OR (Marie-Struempell Disease) OR (Marie Struempell Disease) OR (Rheumatoid Spondylitis) OR (Spondylitis, Rheumatoid) OR (Spondylitis Ankylopoietica) OR (Ankylosing Spondyloarthritis) OR (Ankylosing Spondyloarthritides) OR (Spondyloarthritides, Ankylosing) OR (Spondyloarthritis, Ankylosing) OR (Spondyloarthritis) OR (axial SpA) OR (axial Spondyloarthritis) OR (axSpA) OR (Ankylosing Spondylitis)):ti,ab,kw (Word variations have been searched)

#3 #1 OR #2 (Word variations have been searched)

#4 MeSH descriptor: [C-Reactive Protein] this term only

#5 (C-Reactive Protein) OR (C Reactive Protein) OR (hsCRP) OR (High Sensitivity C-Reactive Protein) OR (High Sensitivity C Reactive Protein) OR (hs-CRP) OR (CRP) (Word variations have been searched)

#6 #4 OR #5 (Word variations have been searched)

#7 MeSH descriptor: [Magnetic Resonance Imaging] this term only

#8 (Magnetic Resonance Imaging) OR (Imaging, Magnetic Resonance) OR (NMR Imaging) OR (Imaging, NMR) OR (Tomography, NMR) OR (Tomography, MR) OR (MR Tomography) OR (NMR Tomography) OR (Steady-State Free Precession MRI) OR (Steady State Free Precession MRI) OR (Zeugmatography) OR (Imaging, Chemical Shift) OR (Chemical Shift Imagings) OR (Imagings, Chemical Shift) OR (Shift Imaging, Chemical) OR (Shift Imagings, Chemical) OR (Chemical Shift Imaging) OR (Magnetic Resonance Image) OR (Image, Magnetic Resonance) OR (Magnetic Resonance Images) OR (Resonance Image,

Magnetic) OR (Magnetization Transfer Contrast Imaging) OR (MRI Scans) OR (MRI Scan) OR (Scan, MRI) OR (Scans, MRI) OR (Tomography, Proton Spin) OR (Proton Spin Tomography) OR (fMRI) OR (MRI, Functional) OR (Functional MRI) OR (Functional MRIs) OR (MRIs, Functional) OR (Functional Magnetic Resonance Imaging) OR (Magnetic Resonance Imaging, Functional) OR (Spin Echo Imaging) OR (Echo Imaging, Spin) OR (Echo Imagings, Spin) OR (Imaging, Spin Echo) OR (Imagings, Spin Echo) OR (Spin Echo Imagings) OR (MRI) (Word variations have been searched)

#9 #7 OR #8 (Word variations have been searched)

#10 #3 AND #6 AND #9 (Word variations have been searched)

188results 5 reviews

Combination：669 papers.

222 deplicates.

Clinical trial registration number:

van der Heijde 2014: NCT00939003.

Anja 2014: NCT00844142 (Trial 1); NCT00235105 (Trial 2)

Maksymowych 2016: NCT01258738

Braun 2016: NCT00265083

Baraliakos 2005: Not available.

Braun 2012: NCT00265083.

Lambert 2007: NCT00195819.

Machado 2012: Not available.

Maksymowych 2010: Not available.

Rudwaleit 2008: Not available.

Song 2011: NCT00844142

Visvanathan 2008: Not available.

This meta-analysis has provided an explicit methodology being addressed with reference to the PICOS strategy:

1) Participants: patients with axial spondyloarthritis;

2) Interventions: inflammation evaluated by MRI-based disease activity score;

3) Comparisons: the level of C-reactive protein concentration;

4) Outcomes: the correlations between C-reactive protein and MRI-detected inflammation;

5) study design: including cross-sectional studies, clinical trials, cohort studies, and randomized controlled trials.
